# “We need to build a better bridge”: findings from a multi-site qualitative analysis of opportunities for improving opioid treatment services for youth

**DOI:** 10.1186/s12954-022-00623-7

**Published:** 2022-04-17

**Authors:** Kirsten Marchand, Oonagh Fogarty, Katrina Marie Pellatt, Kayly Vig, Jordan Melnychuk, Christina Katan, Faria Khan, Roxanne Turuba, Linda Kongnetiman, Corinne Tallon, Jill Fairbank, Steve Mathias, Skye Barbic

**Affiliations:** 1 Foundry, 915-1045 Howe Street, Vancouver, BC V6Z 2A9 Canada; 2 Department of Occupational Science and Occupational Therapy, University of British Columbia, 317-2194 Health Sciences Mall, Vancouver, BC V6T 1Z3 Canada; 3 Faculty of Medicine, University of British Columbia, 317-2194 Health Sciences Mall, Vancouver, BC V6T 1Z3 Canada; 4 Centre for Health Evaluation Outcome Sciences, 588-1081 Burrard Street, Vancouver, BC V6Z 1Y6 Canada; 5 Improving Treatment Together Project, Canadian Centre on Substance Use and Addiction, 75 Albert Street, #500, Ottawa, ON K1P 5E7 Canada; 6 Canadian Centre on Substance Use and Addiction, 75 Albert Street, #500, Ottawa, ON K1P 5E7 Canada; 7 CRISM Prairie Node, Canadian Research Institute on Substance Misuse, 11405-87 Avenue, Edmonton, AB T6G 1C9 Canada; 8 Faculty of Social Work, University of Calgary, 2500 University Drive NW, Calgary, AB T2N 1N4 Canada; 9 Providence Research, 10th floor - 1190 Hornby Street, Vancouver, BC V6Z 1Y6 Canada; 10 St. Paul’s Hospital, Providence Health Care, 1081 Burrard Street, Vancouver, BC V6Z 1Y6 Canada

**Keywords:** Youth, Adolescents, Young adults, Opioid use, Opioid use disorder, Harm reduction, Opioid agonist treatment, Community-based participatory research, Co-design, Qualitative analysis

## Abstract

**Background:**

Adolescence and young adulthood is an important period for substance use initiation and related harms. In the context of the ongoing opioid crisis, the risks for youth (ages 16–29) who use opioids are particularly heightened. Despite recommendations to adopt a developmentally appropriate and comprehensive approach to reduce opioid-related harms among youth, data continue to show that youth are not adequately engaged in opioid treatments and encounter many barriers. The aim of this study is to identify youth-centered opportunities for improving opioid treatment services.

**Methods:**

This paper reports multi-site qualitative findings from youth participating in the ‘Improving Treatment Together’ project, a community-based participatory project being conducted in British Columbia and Alberta, two western Canadian provinces that have been dramatically impacted by the opioid crisis. Qualitative data were collected during three workshops with youth who used opioids and accessed opioid treatment services in the prior 12 months. These workshops were conducted in three communities following the core elements of human-centered co-design. A multi-site qualitative analysis was conducted to identify within- and between-site themes surrounding youths’ needs for improving opioid treatment service experiences and outcomes.

**Results:**

Three overarching needs themes were identified from across the communities. The first reflected youths’ difficulties finding and staying connected to opioid treatment services, with the overarching need theme suggesting opportunities to reduce organizational and systems-related barriers to care, such as waiting times and wider information about service availability. The second area of need was rooted in youths’ feelings of judgment when accessing services. Consequently, opportunities to increase respectful and empathic interactions were the overarching need. The final theme was more nuanced across communities and reflected opportunities for an individualized approach to opioid treatment services that consider youths’ unique basic safety, social, and health needs.

**Conclusions:**

This study identifies fundamental directions for the operationalization and implementation of youth-centered opioid treatment services. These directions are contextualized in youths’ lived experiences accessing services in their local communities, with overarching themes from across sites strengthening their transferability to other settings.

**Supplementary Information:**

The online version contains supplementary material available at 10.1186/s12954-022-00623-7.

## Background

Adolescence and young adulthood is an important period for substance use initiation and risk of related harms [1–6]. As substance use trends among youth (ages 16–29) fluctuate with broader contexts (e.g., drug supply, social norms) [6], non-medical prescription opioid use and illicit opioid use among youth has become a critical public health issue [4]. This is particularly true for youth in the United States (U.S.) and Canada, which have the highest estimated global consumption of opioids, prevalence of opioid use disorder (OUD), and rate of opioid-related drug toxicity deaths [7]. As both countries grapple with drug poisoning crises involving highly potent and contaminated opioids, youth who use opioids are at significant risk of harm, including hospitalization and fatal and non-fatal drug toxicity events [8–11].

To mitigate these opioid-related harms, experts recommend that youth have access to the full continuum of evidence-based interventions through a comprehensive, developmentally appropriate, and recovery-oriented approach [12–15]. These evidence-based interventions can include non-pharmacological treatments, such as counseling, case management, peer support, and person-centered services [12–14]. Pharmacological treatments with opioid agonist treatment (OAT) (e.g., buprenorphine, methadone) are also recommended as they are effective in reducing cravings and withdrawal and increasing treatment retention [12–14]. Harm reduction programs (e.g., safer drug use information and interventions; drug checking; supervised consumption sites [SCSs]) may also minimize the adverse impacts of opioid use and provide an opportunity to deliver additional health and social services and encourage treatment entry [12–14, 16].

Despite these recommendations, recent evidence shows that youth are less likely to access and be retained in these interventions compared to older age groups [17–22]. For instance, health claims data in the U.S. have shown that only 2% of youth received pharmacological treatments within 30 days of a non-fatal drug toxicity event, 29% received non-pharmacological treatments, and an alarming 69% received no treatment [17]. Examining age-related differences in pharmacological treatment retention among people with OUD, Mintz et al. [20] found that 18% of adolescents and 25% of young adults were retained for 6-months compared to 33% of older adults. This research reveals very worrisome gaps in the delivery of opioid treatment services for youth at a time of immense need.

Understanding the reasons for these gaps is critical to ensure that youth have timely access to the full continuum of evidence-based interventions. There are a number of social structural explanations for these gaps, which operate at multiple levels and reflect the developmental and social position of youth compared to adults. For instance, at a policy-level, youth may encounter barriers to treatment and harm reduction interventions due to age-related program restrictions, which often use the biological age of 18. These age-based criteria limit youths’ autonomy in treatment decision-making and increase fears of stigma and law enforcement [18, 23–29]. There are also differences in the social norms of substance use between youth and adults [18, 26]. For example, ethno-epidemiological data suggest that youth tend to obtain substance use equipment from their peers rather than harm reduction programs and are less connected to large networks of street-based substance users [26]. Such differences affect youths’ knowledge of harm reduction practices and engagement in these services.

A number of studies have also discussed service delivery-related barriers that youth encounter when trying to access evidence-based interventions, including OAT. Examples of such barriers include the limited availability of specialists, service provider’s competency in the delivery of OAT to youth, as well as treatment costs and travel requirements [16, 22, 26, 30, 31]. A growing body of evidence has also revealed that youth face individual-level barriers to interventions that arise from a lack of opioid treatment services that are centered on youths’ individual needs and preferences (hereafter referred to as ‘youth-centered’ services) [3, 23–25, 32]. For instance, youth have discussed how long-term OAT does not align with their views of achieving an improved quality of life [32]. Youth have also emphasized the importance of having autonomy in treatment decision-making (e.g., choice of medication, dosages, titration) [32] and their need for a better understanding of the continuum of evidence-based treatments to make such informed decisions [3].

Collectively, this body of evidence underscores that the existing interventions across this continuum are not meeting youths’ needs and preferences [12, 16, 18, 27]. To rectify this, it has been emphasized that the development of youth-centered interventions be co-designed with youth who are using opioids [18, 33, 34]. Accordingly, the ongoing multi-phase and multi-site ‘Improving Treatment Together’ (ITT) project [35] was designed to understand youths’ needs for improving opioid treatment services (Phase 1), and, subsequently, to co-design, implement, and test youth-centered innovations in opioid treatment services (Phase 2–4). The present manuscript reports on the Phase 1 qualitative findings with youth in two western Canadian provinces. As a multi-site study, the findings identify actions that are both community-specific and overarching across sites [36]. Thus, the study contributes a deeper understanding of the contextual factors that influence youths’ local needs, while strengthening the transferability of findings to other communities.

## Methods

### Design and setting

The ITT project follows a community-based participatory research (CBPR) approach [37, 38] and integrates elements of human-centered co-design [39, 40]. Further details about the project’s design and planned methods have been published elsewhere [35]. Briefly, the project is being conducted in British Columbia (BC) and Alberta (AB), two provinces in Canada that have been dramatically impacted by the opioid crisis [8]. Phase 1 of the project occurred between November 2019 and May 2020; Phases 2–4 are ongoing until May 2022. The guiding research question for Phase 1 was: *what are youths’ experiences accessing opioid treatment services and needs for improving these services?*

Following its CBPR approach, the project was launched by a national non-profit organization in partnership with two provincial organizations who support the development and delivery of opioid treatment services for youth. These partners then carried out environmental scans to collaboratively identify four communities that had a high need for opioid treatment services, including OAT, as shown by surveillance data of opioid-related mortality and morbidity among youth [41, 42]. A further goal of the environmental scan was to identify geographically diverse communities that differed in population size (e.g., small, medium, and large urban population centers) [43, 44].

This resulted in the selection of two communities in each province. In BC, the first community (‘BC Interior Community’) was a medium-sized population center (population: 127,380), where, at the time of the scan, the rate of opioid-related overdoses was 17.3 per 100,000 [42]. The second BC community (‘BC Urban Community’) was a large urban center (population: 631,486) where people who use opioids experience high rates of poverty, precarious housing, and where the rate of opioid-related overdoses was 22.9 per 100,000 [42]. In AB, the first community was a medium-sized population center (population: 63,166) in the northern region (‘AB Northern Community’) where the rate of opioid-related overdoses was 15.9 per 100,000 [41]. The final community in AB (‘AB Urban Community’) is one of the most populous urban centers in Western Canada (population: 1,239,220), where opioid-related overdoses were occurring at a rate of 13.9 per 100,000 [41].

The availability of evidence-based interventions is variable across these communities, but generally includes some degree of harm reduction services (e.g., education, injecting and smoking supplies, naloxone distribution, SCSs), OAT (primarily with buprenorphine and methadone), non-pharmacological interventions (e.g., case management, counseling), and detoxification and residential treatment [45]. These programs can be accessed through community-based public programs or private providers/centers (particularly residential and counseling-based treatments). The BC Interior, AB Northern and AB Urban communities deliver some of these interventions through mobile services due to their larger geographical land areas. Meanwhile, the BC Urban Community has been at the forefront of decades of innovative harm reduction and treatment initiatives, such as SCSs [46], injectable OAT [47], and, more recently, safer supply initiatives [48], largely concentrated in the downtown core.

### Sampling and data collection

A total of four workshops were planned for Phase 1, with one per each of the four communities. For each workshop, the target sample included 8–10 youth (i.e., a total of 32–40 youth across workshops) between the ages of 16 and 29 who used opioids (illicit opioids and/or pharmaceutical opioids without prescription) and accessed/received opioid treatment services (e.g., OAT, counseling, case management) in the past 12 months. Further eligibility criteria included ability to provide fully informed consent to participate and speak/write in English. Youth were recruited with the support of the project’s youth peer researchers, community-based partners, and other local organizations who distributed recruitment information. Interested youth then contacted project team members to confirm self-reported eligibility criteria and obtain further details about the workshops.

Upon arrival to each workshop, youth provided fully informed consent to participate and completed a brief questionnaire regarding socio-demographic characteristics, opioid use, and opioid treatment history. After co-creating a safe space agreement, participants separated into smaller discussion groups (akin to focus groups) of 4–6 youth per group to encourage more in-depth discussion and ensure each participant had equal opportunity to contribute to the discussion. Each small discussion group also included trained facilitators and a youth peer-researcher with lived/living opioid use history.

The small group discussions were structured around the first two core elements of human-centered co-design: (1) empathy—aiming to understand youths’ experiences during their point-of-care interactions with opioid treatment services; and (2) identifying needs—aiming to articulate and prioritize the root problems or needs based on their experiences [35, 49, 50]. For both of these elements, participants spent the first 10–15 min individually reflecting on their experiences/needs using facilitation tools that are commonly used in human-centered design (e.g., Journey and/or Empathy Maps [35]). The facilitators then guided participants through a small group discussion for each topic (lasting approximately 1 h each). During small group discussions, participants openly shared, discussed, and elaborated on their unique and common experiences/needs. During these discussions, the facilitators listened actively and supported the conversation by asking clarifying questions (similar to qualitative interviewing). These small group discussions were audio-recorded and included extensive documentation using worksheets, flip charts, and field notes. The total duration of the empathy and needs discussions ranged from 2 to 3 h, and participants were provided with a $75–$125 honorarium (depending on workshop length), snacks/meals, and transportation reimbursement.

As both CBPR and human-centered co-design are meant to adapt to local context and participant needs, several procedures were modified from the initial protocol [35] due to foreseen and unforeseen circumstances. The first workshop was done in the BC Interior Community (November 2019) and provided an opportunity to pilot and refine recruitment and data collection procedures. In this community, we relied primarily on advertisements in collaborating organizations, which resulted in only *n* = 1 participant attending the workshop. For the remaining workshops, we engaged the project’s youth peer-researchers to enhance our outreach strategy. During this first workshop, we also piloted two facilitation tools commonly used in human-centered co-design—the Journey Map and Empathy Map [35]. The remaining three workshops used the Empathy Map only as it was less time consuming while prompting the most in-depth discussion. For the workshop in the BC Urban Community (February 2020), local partners recommended that the socio-demographic questionnaire be collected at the end of the workshop to reduce barriers to participation. Unfortunately, this resulted in a high number of missing questionnaires, precluding the inclusion of the questionnaire data for this site. The workshop in the AB Northern Community took place in February 2020 as originally planned. The workshop in the AB Urban Community was delayed from March to May 2020 and conducted virtually due to the emergence of the COVID-19 pandemic.

### Data analysis

The audio-recordings from the small discussion groups were typed out word for word (i.e., transcribed verbatim), and then transferred to NVivo (version 12, [51]) software for qualitative analysis. A multi-site qualitative analysis (MSQA) [36] was used to identify inductive within- and between-site themes [52]. Due to the small sample size in the BC Interior Community (*n* = 1), the analysis focused on the other three communities.

Based on extensive training and experience in qualitative health services research, authors KM and OF led the analysis. During the first within-site analysis, a semantic and data driven approach was followed to generate initial codes and to search for and define potential themes within each site [52]. Separate meetings were then held with each community’s site-specific team to discuss the data, refine theme coherence, and select supporting quotes. These meetings included youth peer-researchers with lived/living experience, workshop facilitators, and site leads to strengthen member checking, peer debriefing, and collaborative decision-making on the key findings from each site. Subsequently, authors KM and OF began the between-site analysis by independently studying the site-specific theme summaries for their similarities/differences. Extensive discussions led to the development of overarching themes that reflected points of connection across the three communities. The second within-site analysis re-analyzed each community’s transcripts using the overarching themes as a guiding question of the coded data. This stage of the analysis focused on describing the overarching theme’s representation in each site. At this time, the full team met to finalize the overarching themes and their within-site properties. As a final step, these overarching themes were checked for fit with the *n* = 1 youth who participated in the BC Interior Community’s workshop [53].

## Results

A total of 23 youth participated across the three Phase 1 workshops—11 youth in the BC Urban Community; 8 youth in the AB Northern Community; and 4 youth in the AB Urban Community. As noted above, socio-demographic characteristics were not available for BC Urban Community. However, participants who attended the workshop were predominantly recruited through two organizations that provide low-barrier services to inner city youth experiencing homelessness, substance use, and mental health challenges. Socio-demographic data from the two workshops in AB are displayed in Table 1.

**Table 1:** Characteristics of youth participating in AB Northern and AB Urban Community workshops (*n* = 12)

Characteristics	AB Northern and AB Urban Community workshop participants (*n* = 12)^a^
Socio-demographic	Mean ± SD; *N* (%)
Age	24.86 years ± 2.78
*Gender identity:*^*b*^	
Woman	7 (77.8)
Man	2 (22.2)
*Ethnicity:*^*c*^	
Indigenous	1 (11.1)
Indigenous and Caucasian/White	1 (11.1)
Caucasian/White	7 (78.8)
*Highest education level:*^*d*^	
Some high school	5 (45.5)
High school diploma	5 (45.5)
Some university	1 (9)
*Current housing situation:*^*e*^	
Living in apartment with or without roommate(s)	9 (75)
Living with parent/guardian(s)	2 (16.7)
Renting a room	1 (8.3)
*Currently spending most of time in activities:*^*f*^	
Employment	6 (50)
School	2 (16.7)
Volunteering	5 (41.7)
Other activities (e.g., arts, movies, exercise)	7 (58.3)
*Opioid and other substance use patterns*	
Ever used heroin	8 (66.7)
Ever used fentanyl	10 (83.3)
Ever used other non-prescribed or illicit opioids	8 (66.7)
Used heroin in past 12 months	2 (16.7)
Used fentanyl in past 12 months	4 (33.3)
Used other non-prescribed or illicit opioids in past 12 months	1 (8.3)
Used any non-prescribed or illicit opioid in past 12 months	5 (41.7)
Ever used other illicit substances (e.g., cocaine, crystal meth, hallucinogens, etc.)	12 (100)
Used other illicit substances in past 12-months (e.g., cocaine, crystal meth, hallucinogens, etc.)	7 (58.3)
*Opioid use treatment and services in past 12 months*^*f*^	
Opioid agonist treatment	5 (41.7)
Addictions medicine	4 (33.3)
Psychiatry services	4 (33.3)
Clinical counseling services (e.g., CBT, DBT)	7 (58.7)
Case management services	3 (25)
Peer support services	7 (58.7)
Harm reduction services	2 (16.7)

^a^Due to small sample sizes and similarities in the characteristics of participants between communities, descriptive data for the two communities with completed socio-demographic data have been aggregated

^b^Response options included: woman; man; non-binary; two spirit; trans-female; trans-male; not sure/questioning; prefer not to answer; and I don’t identify with any of these. Gender is missing for *n* = 3 participants

^c^Response options included: South Asian; Black/African; Caribbean; Hispanic/Latino; First Nations/Metis/Inuit; Middle Eastern/North African; and Caucasian/White. Participants could choose more than one response option. Ethnicity is missing for *n* = 3

^d^Response options included: Some high school; High school diploma; Some college, technical school education or other certificates/training; Some university education; College or technical degree or diploma; Bachelor’s degree; Master’s degree or higher; Prefer not to answer. Education is missing for *n* = 1

^e^Response options included: homeless; couch surfing; living in single room occupancy hotel; live with parent(s)/guardian(s); live in apartment (independently or with roommates); none of the above; prefer not to answer

^f^Participants could choose more than one response from this set of response options

**Fig. 1:**
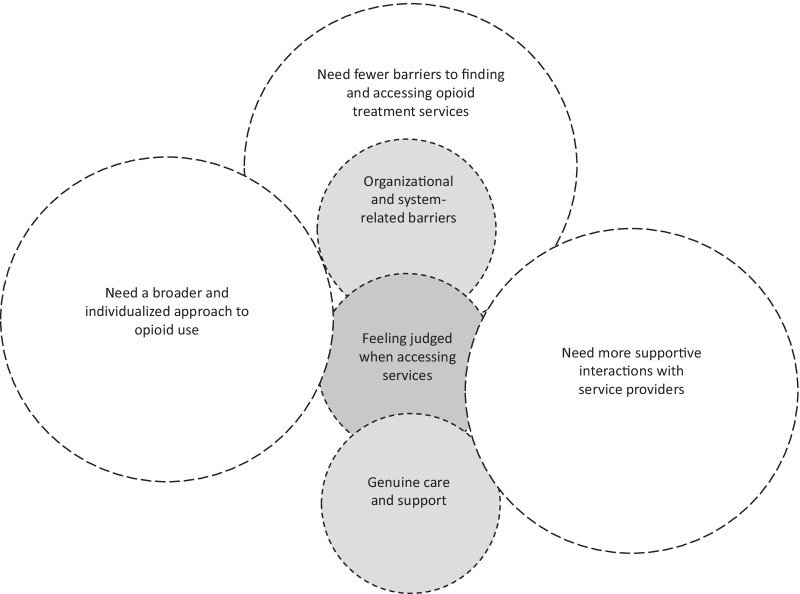
Thematic diagram of main experiences and needs themes from across communities. Venn diagram shows the main experiences (gray circles) and needs (white circles) themes from across the three workshops in the BC Urban, AB Northern, and AB Urban communities. The overlap between the circles illustrates points of connection between the experiences and needs themes

Six overarching experiences and needs themes were identified across communities and are displayed in Fig. 1, with further detail regarding the site-specific sub-themes presented in Table 2 and Additional file 1. As shown in Fig. 1, there was a very strong connection between the overarching experiences and needs themes. For instance, across communities, participants experienced multiple barriers to finding and staying in opioid treatment services, and thus, the need for fewer barriers was an overarching theme. To simplify the presentation of the six overarching themes, the next sections focus on the needs themes, with the experiences themes integrated as supporting explanation. As a MSQA analysis, the needs themes are described separately for the three communities.

**Table 2:** Overarching experiences and needs themes and community-specific sub-themes

Main experiences themes	Sub-themes from each site^a^		
	BC Urban Community	AB Northern Community	AB Urban Community
Organizational and systems-related barriers to opioid treatment services	Obstacles to accessing and staying in services	Obstacles within opioid treatment services	‘Barriers to getting help when I need it’
Feeling judged when accessing services	Being degraded when trying to seek help	Judgment and discrimination from service providers	‘Being seen as a lost cause’
Genuine care and support	‘Feeling like we actually matter’	‘Having legitimate supports to cheer you on’	–
*Main needs themes*			
Fewer barriers to finding and accessing opioid treatment services	Programs should be easier to find and keep connected to, regardless of age	Make treatment access a less overwhelming process	Getting the help I want, when I need it, right at the moment
More supportive interactions with service providers	Service providers who are understanding and make us feel like we actually matter	Service providers who are rooting for me and have been here before	–
A broader and individualized approach to opioid use	Fundamental safety, health, and social necessities to be addressed	Treatment plans should be molded to us and able to change with us	Broader understanding of opioid use and multiple services because there is no one size fits all

^a^Semantic sub-themes were developed for each site. Where possible, these themes are named using direct quotes from workshop participants to closely reflect the theme characteristics in each site

### Community-specific themes in the BC Urban Community

#### Programs should be easier to find and keep connected to, regardless of age

Youth experienced difficulties finding and keeping connected to services, despite the BC Urban Community having a wide range of community-based harm reduction and substance use treatment services for people with OUD. This was primarily due to lengthy waiting times, “aging out” of services at age 24, and getting turned away or kicked out of services. Lengthy waiting times were encountered in multiple contexts, including *“six months of transition to get set up with new care providers”* during the aging out process. Youth also frequently described *“three to four hours waiting for a script [i.e., OAT prescription] while dope sick”* in community-based OAT clinics. Waiting for services was also difficult as youth encountered environments (particularly hospitals and drop-in services) that were uncomfortable (e.g., grimy, cold, not confidential, crowded). These experiences resulted in lost connections to trusted service providers, leaving services without treatment, and for one participant, “*relapsing and stopping my methadone because I didn’t get it [on time] on Friday”*.

To reduce these barriers, youth stressed the need for organizations to provide longer service delivery hours (i.e., evenings, weekends, holidays) and create more comfortable (e.g., low lighting, blankets, chill rooms), and confidential spaces. Youth also discussed that they should be provided with support regardless of their age: *“You should never get turned away from anything… It should just be no matter your age and whatever, if you're an addict [person who uses substances], there should be treatment services for addiction as a whole. And like whether you're 30 or 16, you should be able to go and get the treatment and then be able to access that no matter your age or your situation or your race or your sexuality, right? It shouldn't matter.”*

#### Service providers who are understanding and make us feel like we actually matter

Youth in the BC Urban Community described *“a broad spectrum”* of interactions with service providers, including some *“who are really supportive… and others that are really condescending, or like really hard on you”*. Experiences of judgment were common and salient, as stated by one youth—*“when I come across someone who is kind and considerate, sadly it’s kinda like a shock… they seem like a saint or like an angel”*. These interactions led to feelings of shame and disconnection from services. In contrast, when youth encountered providers who made them *“feel like we actually matter”* (e.g., staying beside youth while going through precipitative withdrawal on Suboxone®), they gained trust, persevered through treatment challenges, and believed more in themselves.

To increase those supportive and respectful interactions with providers, youth stressed the *“need to build a better bridge, a better understanding of how to approach addicts [people who use substances]”.* To build this bridge, youth wanted to interact with providers who were understanding and knowledgeable about opioid use and best practices. Ideally, this knowledge would come through the widespread integration of peer support into opioid treatment services, as youth strongly emphasized their preference for interacting with others who were *“in recovery, who have experience with what we’re going through”*. In the absence of such expertise, youth suggested that organizations *“have your staff that haven’t had that lived experience, you know, tell them… [to] go out and do, you know, 50 h of community service on the Eastside”* (a neighborhood in the BC Urban Community that has a high density of harm reduction services). Youth also strongly expressed their own desire to take on such roles, to give back, and because *“that hour that you’re busy is an hour you didn’t use, and that’s an amazing thing”*.

#### Fundamental safety, health, and social necessities to be addressed

Youth described individual safety, health, and social necessities as a critical priority when seeking opioid treatment services. Ample research among youth using substances in the BC Urban Community has shown the immense challenges they face finding safe and inclusive services to address their needs (e.g., [24, 54–56]). To meet their safety needs, youth called for an expansion of SCSs, drug testing, injectable OAT, and a safer supply of opioids, which they defined as *“clean, lab made and tested drugs”* that would allow them to *“dose properly and safely*”.

Youth also emphasized their need for increased basic social support, including higher monthly disability and income assistance and crisis loan programs, as current supports were *“barely enough to go and live off of”*. A prominent sub-theme reflected youths’ need for *“livable housing”*, which was defined as safe, low barrier, and comfortable (i.e., clean, private). Youth also described their need for seasonally appropriate clothing, particularly for those who were experiencing homelessness. Basic health and hygiene needs were also described, including services for laundry, showering, and more comprehensive nutrition, dental, and prescription medication programs. These needs were strongly connected to a sub-theme that reflected youths’ difficulties finding adequate low-threshold services.

### Community-specific themes in the AB Northern Community

#### Make treatment access a less overwhelming process

Youth in the AB Northern Community encountered obstacles to finding appropriate services and when transitioning between different services. These obstacles reflected the limited capacity of the small number of community-based programs (i.e., one harm reduction service via mobile van and one OAT clinic) for this large geographical service delivery area. Key obstacles here included insufficient information about what services were available for opioid use in their community, *“four-week waiting lists”* for services, limited options for youth with concurrent disorders, and inadequate long-term support *“once you are clean”.* Youth emphasized how overwhelming the process of finding and engaging in services was; as one participant said, *“it took me a million times to actually start using that [OAT] program properly… and I would relapse so many times before I actually was like solid on the program”*.

Youths’ needs for removing these obstacles were focused on improving OAT access by ensuring its affordability (people not on income support pay monthly prescription fees), providing better education about OAT for youth and providers (e.g., medication options, titration process), and changing missed dose policies. As one youth said, *“You can’t get cut off because if you get cut off, what’s your option?… If you don’t have the clean time, you’re going to go back to using [non-prescribed/illicit opioids]… That’s all you know.”*

Additional needs themes in the AB Northern Community focused on “*calming environments”* and transportation to facilitate transitions between different programs. As one participant said, *“we need transportation to and from detox places…to and from treatment centers…to appointments and pharmacies too if they don’t deliver”*.

#### Service providers who are rooting for us and have been here before

Youth in the AB Northern Community also discussed their need for respectful and empathic interactions with service providers. This need was grounded in experiences of judgment when accessing services, as one participant described, *“I found it really hard to find an actual counsellor that like I actually could speak to, I wasn’t sort of judged by, or that kind of stuff… I probably went through like 10 free counsellors before I actually found someone who’s decent.”* This “decent” provider was an individual who was well known to many participants in this community and was seen as *“rooting for me… always believing in me… it didn’t matter how many times I fucked up on Suboxone® [trade name for buprenorphine/naloxone], [they] were like ‘this time, this time’”*. Youth discussed that interactions with service providers who *“respect you and your journey…”* were needed to *“… give you some belief in yourself. And it gives you feelings of not being so alone”*.

Relatedly, youth discussed needing more opportunities to engage with people with lived experience in opioid treatment services. These interactions were described as more relatable and encouraging. As one youth said, *“I think like you need to have people who actually have been there… Like you can’t just be someone who’s never even touched pot or something, you know what I mean, and relate to that person, right?… That definitely was a huge thing for me, is having someone who understood a bit, right? I mean, you never fully understand someone’s situation because everyone’s different, but I feel like it definitely makes a huge difference when you have lived experience.”*

#### Treatment plans should be molded to us and able to change with us

Youth in the AB Northern Community discussed their need for opioid use treatment plans to be less *“cookie cutter*” and adapt to their individual needs over time. As discussed by the following two participants:

Participant M: *“I think one of the biggest problems when it comes to like recovery and stuff, is everyone, like, it’s like this set plan, like ‘This is what you have to do. That’s how like it will work for you.’ But it’s not. Everyone’s different… So, I feel like we need a lot more different options out there, like, if that makes sense?… It should be kind of molded to you in a way.”*

Participant L: *“Yeah, and things change. So then your treatment plan should change with you, right?”*

Participants’ need for individualized treatment plans was grounded in their personalized definitions of “recovery”. Examples included recovering from the *“stress of constantly needing dope”,* the *“consequences of active addiction”*, *“starting a new life”,* and to “*find new healthy alternatives and tools to cope.”* Other treatment goals included finding employment, getting medical assessments, and learning how to take care of themselves (e.g., healthy sleep schedules, cooking, budgeting, managing time, etc.).

### Community-specific themes in the AB Urban Community

#### Getting the help I want, when I need it, right at the moment

In the AB Urban Community, youth emphasized their difficulties *“getting the help I need, when I need it”*. Youth attributed these barriers to a lack of follow-up by service providers, referrals to services that they were not eligible for (e.g., for people using stimulants rather than opioids), and limited availability of services for youth under the age of 18. However, the most salient barrier was the long waiting time for services (and, in particular, those that are publicly funded). As explained by one youth, *“I remember applying once and the waitlist on the affordable treatment center was about four months. By the time four months came up, and I got accepted, I changed my mind already. For a youth who is an addict [person who uses substances], or any addict of any age, can I get the help when I want, when I need it, right at the moment? And that is the most crucial part… whether that's treatment or counseling, or that's opioid dependency program [i.e., OAT], or whatever their program… I think immediacy is definitely high priority.”*

To improve the immediacy of services, youth discussed the need to increase the capacity of opioid treatment services for youth under the age of 18, to ensure these services were more widely distributed throughout the AB Urban Community (and its neighborhoods), and to expand knowledge about such services amongst themselves, family members, and service providers. As one youth summarized: *“I do think, yeah, like, just knowing what is available out there. And like knowing just for yourself, having your parents knowing if they're trying to help their kids… And, like, I know, I personally, I only know about one or two things that like youth under 18 can access for addiction services, and like, especially walk in services, like or stuff like that. It's very, very limited… That and more greater [sic] knowledge and just easily accessible to know what is all out there.”*

#### Broader understanding of opioid use and multiple services because there is no one size fits all

Similar to the other communities, youth in the AB Urban Community experienced judgment and *“being seen as a lost cause”* when accessing services. These experiences were attributed to abstinence-based policies and approaches to opioid treatment services. As one youth said, *“Abstinence isn't always an option for everyone. Basically, those groups that are saying… ‘you have to be sober to seven days or 10 days sober, otherwise, you're a lost cause’, that just turns a lot of people off as it is, because some people don't want to quit or they cannot stop using, but they still want to access some sort of help in order to moderate use, or even just feel connected to people instead of being lonely because loneliness is a huge negative factor when it comes to addiction or even possible overdose deaths.”* To reduce the impacts of these policies and approaches, youth emphasized the importance of adopting a broader understanding of OUD and offering multiple approaches, including *“harm reduction or smart recovery, or 12-steps… because there is no one size fits all.”*

Participants also discussed their need for *“holistic support”*, which included services to address social determinants of health (e.g., housing, employment opportunities), physical health, mental health (psychological, psychiatric, counseling), other ‘dependency programs’ (e.g., for alcohol or crystal methamphetamine use), and family support groups. As one youth explained, *“I have some very dire health issues that aren't addressed in some of those programs… So I have to go to different types of treatment approaches, and different counseling approaches as well. Because just one does not address the specific needs that I need to help me in my recovery.”*

## Discussion

Across communities, youth using opioids discussed their experiences trying to access and remain connected to opioid treatment services over time. Youth reflected on the structural barriers they encountered, and the less frequent but outstanding positive interactions with services. As youth shared these experiences, they identified that critical actions are needed to improve the quality of opioid treatment services. While these experiences and opportunities were influenced by the local context, there were also very strong points of connection across the three communities, which will be the primary focus of our discussion.

The most prominent overarching theme reflects the unequivocal need to increase the number and spread of youth-centered opioid treatment services. This need was independent of community-factors, such as size, rate of opioid-related drug toxicity events, and local politics and policies surrounding harm reduction and OAT. The impact of long waits across communities was significant. Waiting for enrolment to new services or even a simple OAT prescription renewal led to treatment interruptions, and in some worst-case scenarios, relapses. Studies with youth outside of British Columbia and Alberta have echoed the burdens of substance use treatment access [57, 58]. For example, a multi-stakeholder (youth, family, service providers, and others) study of substance use treatment services for youth in the province of Ontario identified several issues related to treatment access [57, 58]. These included regional gaps in services, lack of affordable (i.e., publicly funded) programs, waiting times, limited clinical hours (i.e., business hours), and issues with eligibility and transition planning. These longstanding accessibility issues urge a coordinated national and provincial systems-level response that can be simultaneously tailored to local communities [58]. This should also include the collaborative development of national quality indicators (e.g., wait times, operating hours, breadth of services) for youth substance use treatment, with appropriate local resource allocation, and monitoring over time [59].

The additional organizational and systems-related barriers were much more nuanced between communities. For example, in both the AB Northern and AB Urban communities, participants struggled to find services due to a limited number of programs for youth under the age of 18 and a lack of information about treatment availability. In the BC Urban Community, however, youth expressed the difficulties imposed by aging out policies, and thus wanted to be able to maintain connections to trusted services longer-term. Although youth did not expand on their reasons for these preferences, this may be due to their desire for ‘youth-friendly’ service options [26]. Drawing on other research [26], youth have previously discussed that the attitudes of older adults and service providers in harm reduction and OAT environments were aggressive and judgmental due to youths’ age and social position. Another potential explanation of our finding is that youth may prefer services to be matched to their individual circumstances and treatment needs, rather than biological age alone. As youth conveyed in this study, *“everyone’s different"*, and, therefore, youth needed a holistic and individualized approach in services. To meet this need, service providers should consistently cultivate opportunities to co-develop treatment plans with youth, with attention to what services are offered, in what environment, and for how long. These plans could be informed by socio-ecological frameworks, such as those specific to the opioid crisis [60]. This would encourage service providers to discuss how a young person’s individual- and interpersonal-level factors, such as gender, race, co-occurring mental and physical health needs, family and peer relationships, and community context, can be integrated into treatment planning, leading to a holistic approach to care that is tailored to their specific preferences and circumstances.

At a systems- and policy-level, a broader socio-ecological understanding of opioid use could also inform the wider implementation of an integrated, comprehensive, and person-centered approach to services [60–64]. As youth expressed in all three communities, a broader systems-level response would acknowledge that there is *“no one size fits all”* and combine harm reduction, treatment, social services, and peer supports in youth-centered environments to give youth the best chance of having their needs and goals met. However, there are few examples of programs that have been able to fully realize an integrated and comprehensive approach [60, 61]. This may be partly because social structural factors, such as stigma and the criminalization of people who use opioids, still heavily influence the widespread availability of some interventions (e.g., SCSs, injectable OAT) [60, 65, 66].

Indeed, the overarching theme for supportive interactions illustrated how these factors contributed to youths’ feelings of judgment and shame when accessing services, leading some youth to disconnect from services. In contrast, when youth felt like they *“actually mattered”* to a service provider, their self-determination and service engagement increased. Experiences of structural stigma in the context of opioid treatment services are not unique to youth and have been extensively studied (e.g., [67–71]). However, the impact of such experiences on youth during point-of-care interactions requires careful consideration, as it may compound underlying fears of exposing substance use and fears of criminalization [26, 33].

To reduce the frequency and impact of these experiences, youth in all three communities recommended a stronger integration of youth peer support specialists in opioid treatment services, who are individuals with lived/living experience of substance use. Youth stressed how beneficial peer support was to feeling more understood and less judged in services. Of note, a number of youth in the study also expressed their desire to take on such peer support roles to help others and for the benefits it would bring to their own opioid use treatment goals (e.g., reducing opioid use). The benefit of peer support in harm reduction, community-based substance use treatment, and hospital settings is increasingly supported by empirical evidence [55, 72, 73]. In addition to the relational benefits (e.g., reduced stigma, reduced power imbalances) [55, 74], research suggests that peer support may increase the delivery of information about harm reduction and treatment [75] and connection to services [76]. Thus, peer support models may be especially important in overcoming many of the challenges that youth have described regarding their need for more information about available opioid treatment services and preference for youth-friendly environments. While peer support has been implemented in some youth mental health and substance use services, such as integrated youth service models across Canada (e.g., Foundry, Youth Wellness Hubs Ontario) [77], our results suggest that it is not consistently available across the continuum of services for youth using opioids. Therefore, further implementation and research of peer support specialists is needed to determine how to increase uptake of these services in settings such as OAT, detoxification, and residential treatments.

Our descriptive MSQA study provides several systems- and organizational-related directions to improve the delivery of opioid treatment services. A key strength of our study is the use of human-centered co-design throughout the workshops and ongoing phases of the project. This approach is increasingly being used in healthcare to actively engage lived “end-user” expertise into the co-creation of healthcare interventions, services, and products [39, 78]. In our study, this was crucial to identifying potential solutions to address the complex and evolving social, health, services, and policy-related issues that youth encounter. Based on the prioritized needs identified in the present manuscript, our project team is now in the process of co-creating four opioid treatment service innovations with youth (two in each province). Therefore, the present manuscript provides a unique example of how co-design can be combined with other research methodologies (i.e., CBPR, MSQA) to ensure that youth voices remain central through research, service design, and implementation.

Despite the strengths of our methods to co-designing youth-centered opioid treatment service innovations, the methods are more structured than traditional qualitative approaches where data collection and analysis are commonly done simultaneously with the goal of thematic or theoretical saturation. This limited our ability to further probe or follow-up with youth on some findings (e.g., how substance use patterns impact service access) that would have supported more in-depth understanding of some of the themes and the study’s theoretical contributions. It was also not possible to link participant’s socio-demographic characteristics to their small group discussions, which limits our ability to examine how attributes such as gender, race/ethnicity, and opioid use history may have influenced the qualitative findings. There were also several lessons learned in our use of these innovative participatory methods. The most important of these were the benefit of engaging in active recruitment to be able to reach more young people, as well as collaborating with local community-based organizations to ensure workshop procedures were low-barrier.

## Conclusions

This multi-site study suggests that youth using opioids navigate multiple organizational and systems-related barriers when accessing opioid treatment services. The overarching themes from across communities in British Columbia and Alberta strongly converged around the *“need to build a better bridge”* to opioid treatment services. For the youth in this study, this better bridge will be achieved by increasing access and availability to the continuum of opioid treatment services, considering youths’ holistic and individual needs and preferences, and treating youth with respect and empathy. These findings are vital as policy makers and service providers continue to operationalize and implement youth-centered opioid treatment services.

## Supplementary Information

**Additional file 1**. Three tables displaying the within-site sub-themes for each of the three communities (BC Urban Community, AB Northern Community, AB Urban Community).

## Data Availability

The datasets generated and/or analyzed during the current study are not publicly available due to potential for identifying participants, but are available from the corresponding author on reasonable request.

## Abbreviations

OUD: Opioid use disorder
SCSs: Supervised consumption sites
OAT: Opioid agonist treatment
ITT: Improving treatment together project
CBPR: Community-based participatory research
CCSA: Canadian Centre on Substance Use and Addiction
MSQA: Multi-site qualitative analysis
