# Supplementary material for: “We need to build a better bridge”: findings from a multi-site qualitative analysis of opportunities for improving opioid treatment services for youth
Source: Harm Reduct J. 2022 Apr 17;19:37. doi: 10.1186/s12954-022-00623-7 (PMC9013476; doi:10.1186/s12954-022-00623-7)
Supplement: Supplementary file 1 — Additional file 1. Three tables displaying the within-site sub-themes for each of the three communities (BC Urban Community, AB Northern Community, AB Urban Community). [file 12954_2022_623_MOESM1_ESM.docx]

**Table 1. Within-site themes and sub-themes for the BC Urban Community (n=11)**

| **Between-site Experiences Themes:** | **Within-site Experiences Themes:** | **Within-site Sub-themes: ^a^** |
| --- | --- | --- |
| Organizational and systems-related barriers to opioid treatment services | Obstacles to accessing and staying in services | - Waiting for hours is unbearable - Difficulties with aging out process - Getting pushed away from services - Mental health and substance use separated in services - Not enough services for basic needs - Not enough access or information about harm reduction services - Lack of confidentiality when accessing services |
| Feeling judged when accessing services | Being degraded when trying to seek help | - Feeling judged by service providers - Being treated with pity |
| Genuine care and support | ‘Feeling like we actually matter’ | - Feels good when you have someone looking out for you |
| **Between-site Needs Themes:** | **Within-site Experiences Themes:** | **Within-site Sub-themes: ^a^** |
| Fewer barriers to finding and accessing opioid treatment services | Programs should be easier to find and keep connected to, regardless of age | - Need comfortable and safe service environments to keep connected - Need better transitions or no aging out to keep connected - Need longer service delivery hours - Need services to never turn us away |
| More supportive interactions with service providers | Service providers who are understanding and make us feel like we actually matter | - Need non-judgmental support from service providers - Create more opportunities for youth and other people with lived/living experience to provide peer support in services - Service providers need more education and to be informed about youth and opioid use |
| A broader and individualized approach to opioid use | Fundamental safety, health, and social necessities to be addressed | - Need safe, affordable, comfortable, low-barrier housing - Need more harm reduction services (safe supply, drug testing, discreet places to access harm reduction information and supplies) - Need extra support on triggering dates (holidays, “check day” [the day people eligible for government assistance receive their monthly checks]) - Need seasonally appropriate clothing - Need better social and income support that includes coverage for dental care and prescription medications - Need places to shower, do laundry, take care of hygiene |

**Table Notes:**

a) Semantic sub-themes were developed and where possible, these themes are named using direct quotes from workshop participants to closely reflect the theme characteristics in each site.

**Table 2. Within-site themes and sub-themes for the AB Northern Community (n=8)**

| **Between-site Experiences Themes:** | **Within-site Experiences Themes:** | **Within-site Sub-themes: ^a^** |
| --- | --- | --- |
| Organizational and systems-related barriers to opioid treatment services | Obstacles within opioid treatment services | - Difficulties accessing and staying on opioid agonist treatment - Disconnection between mental health and substance use in services does not make sense - Not knowing where to get help - Waiting for services - Not enough resources once you have stopped using opioids |
| Feeling judged when accessing services | Judgment and discrimination from service providers | - Feeling judged and labelled from service providers - Feeling judged for being enrolled in opioid agonist treatment |
| Genuine care and support | ‘Having legitimate supports to cheer you on’ | - Encouragement from service providers - Receiving information about opioids and services in a non-judgmental way |
| **Between-site Needs Themes:** | **Within-site Needs Themes:** | **Within-site Sub-themes: ^a^** |
| Fewer barriers to finding and accessing opioid treatment services | Make treatment access a less overwhelming process | - Need a safe and welcoming service environment - Remove barriers to opioid agonist treatment specifically - Need transportation to services |
| More supportive interactions with service providers | Service providers who are rooting for me and have been here before | - Need empathy and respect from service providers - Need people with lived experience who understand what we have been through |
| A broader and individualized approach to opioid use | Treatment plans should be molded to us and able to change with us | - Need treatment plans to be individualized - Need supports to help with other impacts of opioid use that might be different for each person (e.g., legal, family relationships, employment, housing) - Need harm reduction to be accepted and available in rural areas |

**Table Notes:**

a) Semantic sub-themes were developed and where possible, these themes are named using direct quotes from workshop participants to closely reflect the theme characteristics in each site.

**Table 3. Within-site themes and sub-themes for the AB Urban Community (n=4)**

| **Between-site Experiences Themes:** | **Within-site Experiences Themes:** | **Within-site Sub-themes: ^a^** |
| --- | --- | --- |
| Organizational and systems-related barriers to opioid treatment services | ‘Barriers to getting help when I need it’ | - Encountering programs based on a ‘one size fits all’ approach - Getting turned away from services - Lengthy waiting times - Shortage of youth-specific services |
| Feeling judged when accessing services | ‘Being seen as a lost cause’ | - Hearing that we are a failure or a lost cause - Hiding substance use from service providers - Experiencing shame from service provider |
| Genuine care and support | - |  |
| **Between-site Needs Themes:** | **Within-site Needs Themes:** | **Within-site Sub-themes: ^a^** |
| Fewer barriers to finding and accessing opioid treatment services | Getting the help I want, when I need it, right at the moment | - Need quicker access to publicly funded treatment - Need more knowledge and information about what services are out there - Need services to be available in smaller and rural communities - Need transportation to services - Need more services for youth under the age of 18 |
| More supportive interactions with service providers | - | - |
| A broader and individualized approach to opioid use | Broader understanding of opioid use and multiple services because there’s no one size fits all | - Need services and programs that match youths’ treatment goals - Need harm reduction services and supports - Need other dependency programs (e.g., for crystal methamphetamine) - Need holistic support to be available – social determinants of health, mental health, physical spiritual |

a) Semantic sub-themes were developed and where possible, these themes are named using direct quotes from workshop participants to closely reflect the theme characteristics in each site.
